# Spontaneous neonatal pneumomediastinum with spinnaker-sail sign: A case report

**DOI:** 10.1016/j.radcr.2026.07.134

**Published:** 2026-09-01

**Authors:** Cláudia Ferreira Miguel, Mariana Meneses Oliveira, Teresa Torres, Beatriz Parreira de Andrade

**Affiliations:** a Serviço de Pediatria e Neonatologia, Unidade Local de Saúde de Gaia e Espinho, Vila Nova de Gaia, Portugal; b Unidade de Neonatologia, Serviço de Pediatria e Neonatologia, Unidade Local de Saúde de Gaia e Espinho, Vila Nova de Gaia, Portugal

**Keywords:** Newborn, Pneumomediastinum, Chest radiography, Spinnaker-sail sign

## Abstract

Neonatal pneumomediastinum is defined by the presence of free air within the mediastinum, usually associated with risk factors such as prematurity, meconium aspiration or neonatal resuscitation. Spontaneous pneumomediastinum is a rare condition in newborns. Chest radiography is crucial to the diagnosis and may demonstrate typical patterns, including the Spinnaker-sail sign. We report the case of a term male newborn delivered by uncomplicated vaginal delivery with no need for resuscitation. On day 2 of life, muffled heart sounds prompted echocardiographic evaluation, which was limited by marked interposed gas. Chest radiography subsequently demonstrated pneumomediastinum with the characteristic Spinnaker-sail sign. The newborn was admitted to the Neonatal Intensive Care Unit for monitoring and received low-flow oxygen. He presented a benign clinical course with complete resolution of the air leak by day 7 of hospitalization. This case highlights the importance of careful physical examination and recognition of radiographic patterns of pneumomediastinum in neonates to ensure appropriate diagnosis and management.

## Introduction

Neonatal pneumomediastinum or mediastinal emphysema is an uncommon condition defined as free air in the mediastinum, with reported incidence ranging from 4 to 25 cases per 10,000 live births [1,2]. A retrospective series conducted in a neonatal intensive care unit in Zurich reported a frequency of approximately 0.1% among admitted neonates [3]. The incidence of spontaneous pneumomediastinum specifically remains poorly established.

Neonatal pneumomediastinum is usually associated with prematurity, meconium aspiration, birth trauma, or positive-pressure ventilation, although it may rarely occur without identifiable precipitating factors (spontaneous pneumomediastinum) [1,2,4,5]. The clinical presentation is variable, ranging from asymptomatic newborns to the presence of significant respiratory distress [1]. Nevertheless, most patients follow a benign and self-limiting course [2].

Chest radiography remains the gold standard imaging modality for the diagnosis of neonatal pneumomediastinum [1]. In neonates, the presence of retrothymic air may elevate the thymic lobes from the cardiac silhouette, resulting in a classic Spinnaker-sail sign in thorax radiography. This radiographic sign is highly characteristic and is considered pathognomonic of neonatal pneumomediastinum [6]. We report a case of spontaneous neonatal pneumomediastinum presenting with the characteristic Spinnaker-sail sign.

## Case report

Male newborn born to a 28-year-old healthy mother after a monitorized and uneventful pregnancy. Routine prenatal ultrasonographies were unremarkable, with no fetal thoracic abnormalities identified. Delivery occurred at 39 weeks and 6 days through uncomplicated spontaneous vaginal birth with Apgar 9/10. Screening for critical congenital heart disease on day 1 of life was normal.

**Fig. 1:**
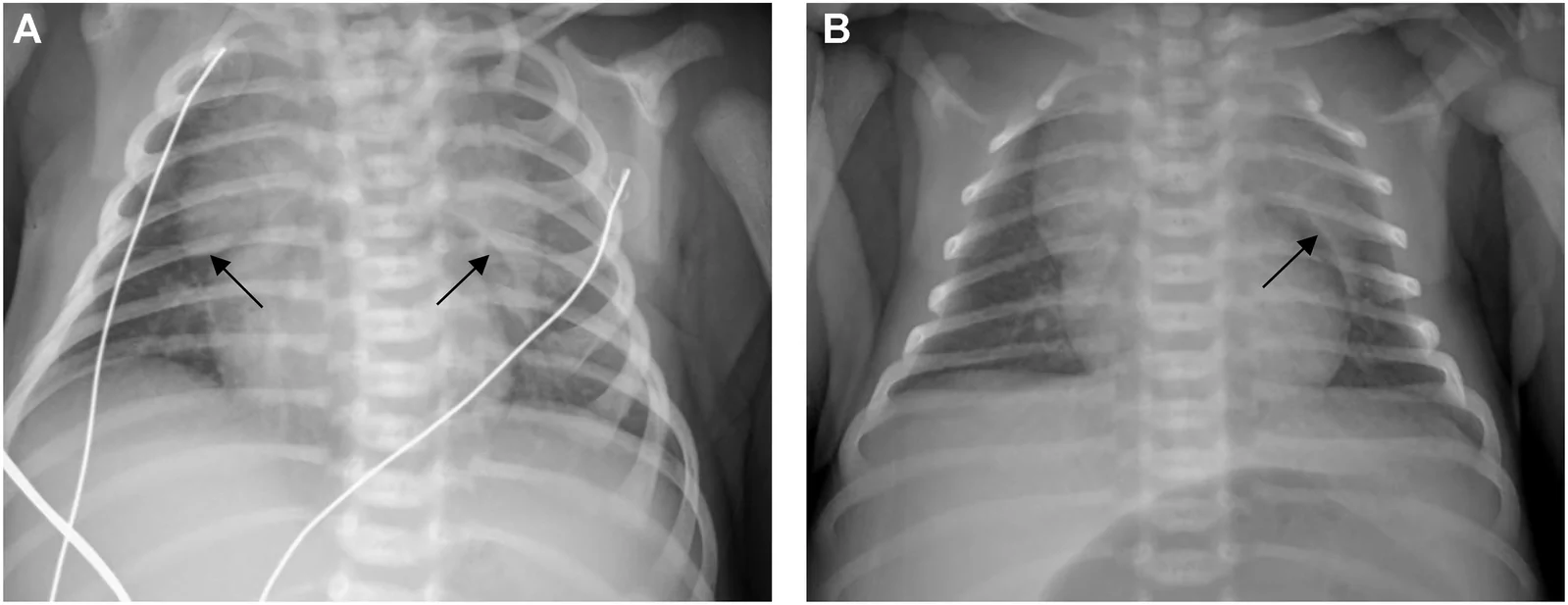
Frontal chest radiographs demonstrating neonatal pneumomediastinum. (A) Mediastinal air elevates the thymic lobes (arrows), producing the characteristic Spinnaker-sail sign. (B) Follow-up radiograph at day 5 of hospitalization showing persistent elevation of the left thymic lobe (arrow).

On the second day of life, clinical evaluation found intermittent tachypnea, and cardiac auscultation revealed muffled heart sounds in an otherwise well-perfused newborn, without other signs of significant respiratory distress. Echocardiographic evaluation was limited by marked interposed gas. A chest radiograph demonstrated the characteristic Spinnaker-sail sign, establishing the diagnosis of pneumomediastinum (Fig. 1). Thoracic ultrasound confirmed pneumomediastinum, with no additional abnormalities, namely pleural effusion, lung consolidation or other associated air-leak syndromes. Laboratory evaluation was unremarkable.

**Fig. 2:**
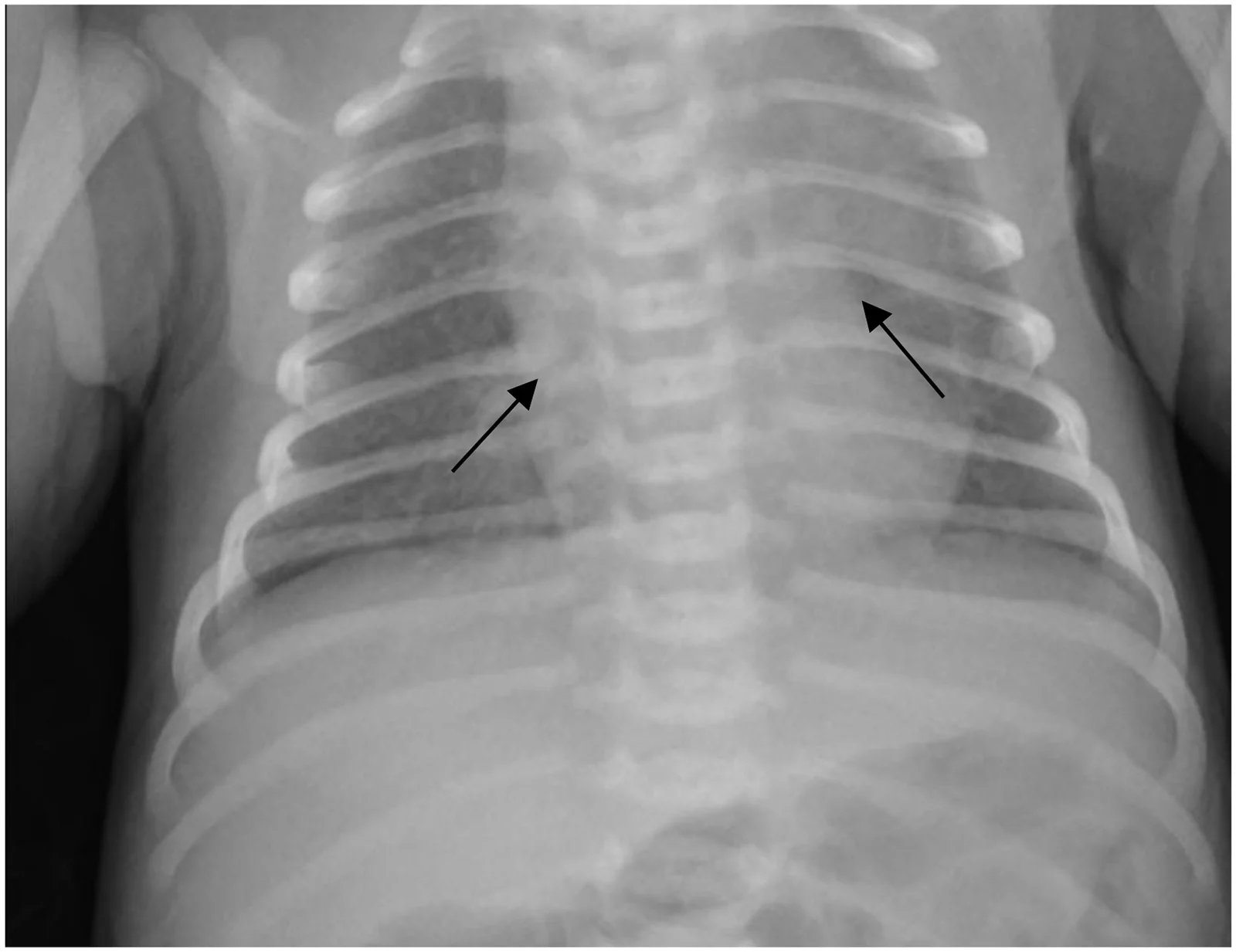
Follow-up chest radiograph obtained on day 7 of hospitalization demonstrating complete resolution of the pneumomediastinum and normalization of thymic contour (arrows).

The newborn was transferred to the Neonatal Intensive Care Unit for clinical monitoring. Supplemental oxygen was administered via nasal cannula, with a maximum flow of 1 L/min, until day 4 of hospitalization. The newborn remained hemodynamically stable throughout admission. Intermittent tachypnea persisted for less than 24 hours, without retractions, cyanosis, or other signs of respiratory distress. Peripheral oxygen saturation remained consistently above 95%. Subsequent follow-up chest radiographs were performed, with complete resolution of the pneumomediastinum by day 7 of hospitalization (Fig. 2). The newborn remains under regular follow-up in the Neonatology outpatient clinic, with no reported complications and no recurrence of pneumomediastinum at 3 months after discharge.

## Discussion

Neonatal air-leak syndromes remain clinically relevant because of their potential for respiratory and hemodynamic compromise [7]. Neonatal pneumomediastinum represents a rare condition, with estimated incidence ranging from 4 to 25 cases per 10,000 live births, although its true incidence may be underestimated considering that mildly symptomatic or asymptomatic cases may remain undiagnosed [2,4].

The pathophysiology of pneumomediastinum is explained by a sudden increase in the pressure gradient between the alveoli and the surrounding interstitium with alveolar rupture, allowing air to enter the pulmonary interstitium and dissect along the peribronchial and perivascular connective tissue sheaths toward the mediastinum [2,8]. Recognized risk factors for this condition include prematurity, meconium aspiration, underlying pulmonary disease, neonatal resuscitation, and traumatic or prolonged delivery, all of which may contribute to this mechanism [4]. In hospitalized newborns, mechanical ventilation with high peak airway pressure and positive end expiratory pressure is a major predisposing factor [9]. On the other hand, spontaneous pneumomediastinum is less common after uncomplicated delivery in otherwise healthy term neonates. In such cases, transient increases in intra-alveolar pressure, for example during vigorous crying, may generate sufficiently high pressure gradients to cause alveolar rupture and air leakage into the mediastinum [2]. Our case resembles previous reports of spontaneous pneumomediastinum in term neonates without identifiable risk factors, like the one described by Mawlana and Osman [10].

The clinical presentation of neonatal pneumomediastinum is variable, ranging from an incidental imaging finding in asymptomatic infants to significant respiratory distress [1,11]. Muffled heart sounds may occur when mediastinal air is interposed between the heart and the anterior chest wall, although this finding is not specific. In our case, intermittent tachypnea and muffled heart sounds were the main clinical abnormalities. A similar presentation was described by Choe et al. [12] who reported spontaneous pneumomediastinum in a healthy term neonate initially detected because of decreased heart sounds during routine physical examination [12]. These cases highlight the potential diagnostic value of careful cardiac auscultation in otherwise well-appearing neonates.

The main radiographic differential diagnoses of neonatal pneumomediastinum include pneumopericardium and pneumothorax, while atypical radiologic findings may prompt consideration of pulmonary interstitial emphysema or congenital thoracic lesions [1,11,13]. Pneumopericardium typically outlines the cardiac silhouette without displacing the thymus, whereas pneumothorax is characterized by a visceral pleural line with absence of peripheral lung markings [2]. In contrast, pneumomediastinal air separates the thymus from the cardiac silhouette, elevating its lobes superiorly and laterally, and creating the Spinnaker-sail sign. Another classic radiographic finding in pneumomediastinum is the “continuous diaphragm” sign, in which gas interposed between the heart and diaphragm outlines the central portion of the diaphragm [13]. In our patient, chest radiography established the diagnosis by demonstrating the pathognomonic Spinnaker-sail sign, thereby avoiding the need for further imaging investigations associated with additional radiation exposure.

In our case, thoracic ultrasound was performed as a complementary bedside examination and confirmed the presence of mediastinal air, while showing no pleural effusion or pulmonary consolidation. Thoracic ultrasound has gained increasing interest in neonatal air-leak syndromes as a bedside, repeatable, radiation-free imaging modality. Nevertheless, distinguishing pneumomediastinum from pneumothorax may be challenging, particularly when different air-leak syndromes coexist, and it remains an operator-dependent examination [14]. Computed tomography may provide further anatomical detail when radiographic findings are equivocal or an underlying structural abnormality is suspected. However, given the ionizing radiation exposure, its use should be carefully considered and reserved for selected cases [2,13].

Management of neonatal pneumomediastinum is generally conservative, as most cases resolve spontaneously, with close cardiorespiratory monitoring and respiratory support provided only when clinically indicated [4,11]. Although its clinical course is usually benign, pneumomediastinum may coexist with other air-leak syndromes and may rarely progress to tension pneumomediastinum, resulting in respiratory or hemodynamic compromise [3,9]. Invasive treatment is rarely required and is reserved for patients with progressive respiratory or hemodynamic compromise, with few patients requiring high-frequency ventilation or invasive drainage [15]. In our patient, low-flow supplemental oxygen was administered via nasal cannula as nitrogen washout therapy, despite oxygen saturation consistently above 95%. However, the use of supplemental oxygen to accelerate air reabsorption in air-leak syndromes remains controversial and, to our knowledge, available evidence evaluating its efficacy in neonates is limited to studies of neonatal pneumothorax case series [16,17].

## Conclusion

Spontaneous neonatal pneumomediastinum is uncommon and may have a subtle clinical presentation, even in the presence of significant radiographic findings. Our case is particularly unusual because the condition occurred in a term newborn without identifiable risk factors. This emphasizes the value of detailed physical examination, as muffled heart sounds were the key clue leading to further investigation in an otherwise well-appearing newborn. Chest radiography remains essential for the diagnosis and the presence of the Spinnaker-sail sign is pathognomonic of pneumomediastinum [18]. Although neonatal pneumomediastinum is usually a benign and self-limiting condition, delayed diagnosis may lead to respiratory compromise, cardiovascular compression, or progression of air-leak syndromes. Rarely, tension pneumomediastinum may develop, resulting in hemodynamic instability and requiring urgent intervention. Therefore, prompt recognition is essential to ensure appropriate monitoring and to avoid unnecessary diagnostic procedures [1].

## Patient consent

Written informed consent was obtained for publication of this case report, any accompanying clinical information and images.

## References

[1] Alhudhaif J.M., Almusallam A.Y., Alfayez A.A., Alqahtani H.S., Alwazzan S. (2022). Benign spontaneous pneumomediastinum in a late preterm newborn. Cureus.

[2] Zamlout A., Jamahiri B., Jabbour E. (2025). Spinnaker-sail sign in full-term neonates with spontaneous pneumomediastinum: a case study and scoping literature review. BMC Pediatr..

[3] Hauri-Hohl A., Baenziger O., Frey B. (2008). Pneumomediastinum in the neonatal and paediatric intensive care unit. Eur J Pediatr.

[4] Rocha G., Guimarães H. (2018). Spontaneous pneumomediastinum in a term neonate: case report. Clin Case Rep.

[5] Hafis Ibrahim C.P., Ganesan K., Mann G., Shaw N.J. (2009). Causes and management of pulmonary air leak in newborns. Paediatr Child Health.

[6] Akin K., Cizmeci M.N., Kanburoglu M.K., Akelma A.Z., Tatli M.M. (2013). Angel wing sign in a neonate with pneumomediastinum. J Pediatr.

[7] Beaton A., Sendi P., Martinez P.A., Totapally B.R. (2024). Prevalence and outcomes of air leak syndrome and subtypes in neonates in the United States. J Neonatal Perinatal Med.

[8] Gray J.M., Hanson G.C. (1966). Mediastinal emphysema: aetiology, diagnosis, and treatment. Thorax.

[9] Mohamed I.S.I., Lee Y.H., Yamout S.Z., Fakir S., Reynolds A.M. (2007). Ultrasound guided percutaneous relief of tension pneumomediastinum in a 1-day-old newborn. Arch Dis Child Fetal Neonatal Ed.

[10] Mawlana W.H., Osman A. (2020). Spontaneous pneumomediastinum in term neonate: a case report. Austin J Clin Case Rep.

[11] Teo S.S.S., Priyadarshi A., Browning Carmo K. (2019). Sail sign in neonatal pneumomediastinum: a case report. BMC Pediatr.

[12] Choe Y.J., Kim E.S., Kim E.K., Kim H.S., Chun J.E., Kim W.S. (2010). Decreased heart sound in a healthy newborn: spontaneous multiseptated cystic pneumomediastinum with delayed respiratory distress. Korean J Pediatr.

[13] Raissaki M., Modatsou E., Hatzidaki E. (2019). Spontaneous pneumomediastinum in A term newborn: atypical radiographic and CT appearances. BJR Case Rep.

[14] Sagaser A.E., Reeves A., Arnautovic T., Sanchez-Esteban J. (2024). Distinction between pneumothorax and pneumomediastinum using point-of-care ultrasound (POCUS): role of still lung point. AJP Rep.

[15] Ogundowole G., Ogonor E., Ojha S., Ruggins N. (2025). Pneumomediastinum in a term neonate: a rare clinical entity. Infant.

[16] Shaireen H., Rabi Y., Metcalfe A., Kamaluddeen M., Amin H., Akierman A. (2014). Impact of oxygen concentration on time to resolution of spontaneous pneumothorax in term infants: a population-based cohort study. BMC Pediatr.

[17] Margaliot A., Mangel L., Waxman Y., Be’er M., Marom R., Herzlich J. (2025). Outcomes of spontaneous pneumothorax in neonates: treatments vs. expectant management. J Perinatol.

[18] Monteiro R., Paulos L., do Agro J., Winckler L. (2015). Neonatal spontaneous pneumomediastinum and the Spinnaker-Sail sign. Einstein (São Paulo).

